# DIGitoxin to Improve ouTcomes in patients with advanced chronic Heart Failure (DIGIT‐HF): Baseline characteristics compared to recent randomized controlled heart failure trials

**DOI:** 10.1002/ejhf.3679

**Published:** 2025-05-19

**Authors:** Udo Bavendiek, Nele Henrike Thomas, Dominik Berliner, Xiaofei Liu, Johannes Schwab, Andreas Rieth, Lars S. Maier, Sven Schallhorn, Eleonora Angelini, Samira Soltani, Fabian Rathje, Mircea‐Andrei Sandu, Welf Geller, Thomas Gaspar, Rainer Hambrecht, Marija Zdravkovic, Sebastian Philipp, Dragana Kosevic, Georg Nickenig, Daniel Scheiber, Sebastian Winkler, Peter Moritz Becher, Philipp Lurz, Martin Hülsmann, Maria von Karpowitz, Christoph Schröder, Barbara Neuhaus, Anika Seltmann, Heiko von der Leyen, Christian Veltmann, Stefan Störk, Michael Böhm, Armin Koch, Anika Großhennig, Johann Bauersachs, the DIGIT‐HF Study Group, Udo Bavendiek, Johann Bauersachs, Christoph Schindler, Armin Koch, Dirk O. Stichtenoth, Udo Bavendiek, Johann Bauersachs, Christian Veltmann, Michael Böhm, Armin Koch, Heiko von der Leyen, Stefan Störk, Stefan D. Anker, Hans J. Trampisch, Paul Mohacsi, Gerhard Pölzl, Anika Großhennig, Maria von Karpowitz, Ulrich Tebbe, Markus Haass, Stephan von Haehling, Robert Stöhr, Nikolaus Marx, Andreas Rieth, Veselin Mitrovic, Andreas Ritzel, Johannes Haas, Frank Edelmann, Tobias Trippel, Sebastian Winkler, Mirko Seidel, Monika Ernst, Christoph Hanefeld, Andreas Mügge, Kristina Becker, Georg Nickenig, Ulrich M. Becher, Johann Auer, Rainer Hambrecht, Harm Wienbergen, Johannes Brachmann, Steffen Schnupp, Ralph Oeckinghaus, Timo Aschenbrenner, Christopher Piorkowski, Thomas Paul Gaspar, Christian Meyer, Kristin Riße, Ralf Westenfeld, Malte Kelm, Constantin von zur Mühlen, Sebastian Grundmann, Kristian Hellenkamp, Tim Seidler, Friedrich Fruhwald, Klemens Ablasser, Alexander Vogt, Sebastian Nuding, Herbert Nägele, Daniel Stierle, Dirk Westermann, Mahir Karakas, Udo Bavendiek, Johann Bauersachs, Lutz Frankenstein, Tobias Täger, Michael Böhm, Ingrid Kindermann, Paul Christian Schulze, Julian Georg Westphal, Roman Pfister, Stephan Rosenkranz, Rolf Wachter, Michael Metze, Marcus Sandri, Holger Thiele, Tobias Graf, Jan‐Christian Reil, Rüdiger Braun‐Dullaeus, Alexander Schmeißer, Thomas Münzel, Tommaso Gori, Bernhard Schieffer, Wolfram Grimm, Jens Taggeselle, Antje Stumpp, Roland Prondzinsky, Susanne Rode, Norbert Schön, Brigitte Schön, Stefan Kääb, Stefan Brunner, Johannes Schwab, Matthias Pauschinger, Andreas Götte, Volker Rickert, Uwe Gremmler, Michael Krumm, Lars Maier, Bernhard Unsöld, Markus Schwefer, Stefan Hettwer, Stefan Rausch, Kyrill Rogacev, Sebastian Philipp, Torsten Lauf, Martin Hülsmann, Stefan Störk, Bettina Kraus

**Affiliations:** ^1^ Department of Cardiology and Angiology Hannover Medical School Hannover Germany; ^2^ Institute for Biostatistics, Hannover Medical School Hannover Germany; ^3^ Department of Cardiology Paracelsus Medical University Nuremberg Germany; ^4^ MVZ Kardiologie, Klinikum Neumarkt Neumarkt Germany; ^5^ Department of Cardiology Kerckhoff‐Klinik Bad Nauheim Germany; ^6^ Department for Internal Medicine II University Hospital Regensburg Regensburg Germany; ^7^ Department of Internal and Cardiovascular Medicine Herzzentrum Dresden, University Clinic, Dresden, Technische Universität Dresden Dresden Germany; ^8^ Department of Internal Medicine II, Cardiology, Angiology and Intensive Care Medicine Klinikum Links der Weser Bremen Germany; ^9^ Clinic for Internal Medicine, University Clinical Hospital Center Bezanijska Kosa, Faculty of Medicine, University of Belgrade Belgrade Serbia; ^10^ Department of Internal Medicine, Cardiology and Intensive Care Medicine Elbeklinikum Stade Stade Germany; ^11^ Department of Cardiology Institute for Cardiovascular Diseases Dedinje Belgrade Serbia; ^12^ Department of Medicine‐Cardiology University Hospital Bonn Bonn Germany; ^13^ Division of Cardiology, Pulmonology and Vascular Medicine University Hospital Duesseldorf, Heinrich‐Heine University Duesseldorf, Medical Faculty Duesseldorf Germany; ^14^ Department of Internal Medicine BG Klinikum Unfallkrankenhaus Berlin Berlin Germany; ^15^ Department of Cardiology University Heart and Vascular Center Hamburg Hamburg Germany; ^16^ German Center of Cardiovascular Research (DZHK), Partner Site Hamburg/Kiel/Lübeck Hamburg Germany; ^17^ University Medical Center Mainz, Center of Cardiology, Johannes Gutenberg University Mainz Germany; ^18^ Universitätsklinik für Innere Medizin II, Abteilung Kardiologie, Medizinische Universität Wien Wien Austria; ^19^ Institute of Clinical Pharmacology, Hannover Medical School Hannover Germany; ^20^ Center for Clinical Trials, Hannover Medical School Hannover Germany; ^21^ Orgenesis, Inc Germantown MD USA; ^22^ Hannover Medical School Hannover Germany; ^23^ Center for Electrophysiology Bremen Bremen Germany; ^24^ Department Clinical Research & Epidemiology, Comprehensive Heart Failure Center Würzburg, and Department Internal Medicine I University Hospital Würzburg Würzburg Germany; ^25^ Klinik für Innere Medizin III, HOMICAREM (HOMburg Institute for CArdioREnalMetabolic Medicine), Universitätsklinikum des Saarlandes, Saarland University Homburg Germany

**Keywords:** Heart failure, HFrEF, Cardiac glycosides, Digitoxin, Randomized clinical trial

## Abstract

**Aims:**

This report presents the baseline characteristics of patients enrolled in the DIGIT‐HF trial and compares them with participants from recent trials with improved outcomes in patients with heart failure (HF) and a reduced ejection fraction (HFrEF).

**Methods and results:**

DIGIT‐HF, a randomized, double‐blind, placebo‐controlled, multicentre trial enrolling patients with symptomatic HFrEF (New York Heart Association [NYHA] functional class II and left ventricular ejection fraction [LVEF] ≤30%, or NYHA class III–IV and LVEF ≤40%), compares the efficacy and safety of digitoxin versus placebo in addition to standard treatment. Most baseline characteristics of the intention‐to‐treat population (1212 patients, mean age 66 ± 11 years, 20% women, mean LVEF 29 ± 7%) were similar to those in recent HFrEF trials. The distribution of NYHA class II, III, and IV was 30%, 66% and 4%, respectively, and indicates that the patients were sicker than in comparator HFrEF trials. Less patients had atrial fibrillation (27%) than those in recent HFrEF trials, but prescription rates of background therapy with beta‐blockers (96%), angiotensin‐converting enzyme inhibitors/angiotensin receptor blockers/angiotensin receptor–neprilysin inhibitors (95%), mineralocorticoid receptor antagonists (76%), and diuretics (87%) were high and similar. Overall, 40% of patients were on angiotensin receptor–neprilysin inhibitors, 19% on sodium–glucose cotransporter 2 inhibitors, and 9% on ivabradine. Rates of implantable cardioverter‐defibrillator (ICD, 64%) and cardiac resynchronization therapy (CRT, 25%) devices were much higher than in recent HFrEF trials.

**Conclusions:**

Patients included in DIGIT‐HF display a more severe HF symptom burden and higher rates of ICD/CRT implants compared to participants in recent HFrEF trials, while pharmacotherapy was largely similar.

Clinical Trial Registration: EudraCT (2013–005326‐38).

## Introduction

Cardiac glycosides have been used in the treatment of heart failure (HF) for two centuries. However, the evidence demonstrating their beneficial effects in HF with reduced ejection fraction (HFrEF) is sparse and mainly based on the DIG trial, the only randomized, placebo‐controlled clinical trial of substantial size. The DIG trial revealed that digoxin exerted neutral effects on the primary endpoint of mortality; however, it indicated a significant reduction of the pre‐defined secondary endpoint hospitalizations for worsening HF in patients with a left ventricular ejection fraction (LVEF) ≤45%.^1^ Subgroup analyses indicated that patients with markedly reduced ejection fractions (<30%) and pronounced HF symptoms (New York Heart Association [NYHA] III–IV) may benefit particularly from the effect of digoxin on outcomes.^2^ Furthermore, low serum concentrations of digoxin (0.5–0.9 ng/ml) were associated with a reduced mortality risk, whereas serum concentrations above 1 ng/ml seemed to worsen the prognosis.^3^, ^4^, ^5^ In the DIG trial, the background HF therapy consisted of angiotensin‐converting enzyme inhibitors (ACEi) and diuretics, only. This is due to the fact that the DIG trial was conducted before clinical trials with beta‐blockers, mineralocorticoid receptor antagonists (MRA), angiotensin receptor–neprilysin inhibitors (ARNI) and sodium–glucose cotransporter 2 inhibitors (SGLT2i) as well as cardiac device therapies (cardiac resynchronization therapy [CRT], implantable cardioverter‐defibrillator [ICD]) had been published, all demonstrating improved outcomes in patients with HFrEF.^6^, ^7^, ^8^

The DIGitoxin to Improve ouTcomes in patients with advanced chronic Heart Failure (DIGIT‐HF) trial was designed to investigate the potential beneficial effects of the cardiac glycoside digitoxin on clinical outcomes in patients diagnosed with HFrEF,^9^, ^10^ in addition to contemporary medical therapy as recommended by current HF guidelines.^6^, ^7^, ^8^ Digitoxin was selected over digoxin on the basis that no prospective, randomized clinical trial investigating the effect of digitoxin on outcomes in HFrEF has been reported, and because of its more favourable pharmacokinetic profile. Due to its compensatory entero‐hepatic elimination, digitoxin enables stable blood concentrations even in patients with developing or advanced renal dysfunction.^11^, ^12^, ^13^ This largely avoids drug concentrations above the effective therapeutic range causing adverse and potential toxic side effects, which are common with digoxin, in particular if prescribed in patients with worsening renal function.^12^, ^13^ These pharmacokinetic advantages of digitoxin over digoxin are important to facilitate a simple trial design without the need for regular controls after achievement of serum target concentrations, which were defined accordingly to the effective narrow ranges of 0.5 to 0.9 ng/ml for digoxin provided in the DIG trial.

Here, we report the baseline characteristics of patients enrolled in the DIGIT‐HF‐trial. In addition, these are compared to study cohorts of three recently performed randomized controlled clinical trials demonstrating improved outcomes in patients with chronic HFrEF to address the similarities and in particular relevant differences of main characteristics among these trials, which will be important for interpretation of the final DIGIT‐HF trial results.

## Methods

DIGIT‐HF is a randomized, double‐blind, parallel‐group, placebo‐controlled, two‐arm, multicentre, event‐driven phase IV trial evaluating the efficacy and safety of digitoxin compared to placebo in addition to standard of care (SOC) in patients with HFrEF. The trial is registered as EudraCT number 2013‐005326‐38, and its design has previously been published.^9^, ^10^ The study was approved by the respective national competent authorities as well as ethics committees, and was conducted according to current Good Clinical Practice.

### Patients

Patients with systolic chronic HF NYHA functional class III–IV and LVEF ≤40% or NYHA class II and LVEF ≤30% who are at least 18 years of age were evaluated for trial participation at the screening visit. Following the provision of written informed consent and the completion of the screening visit, patients who met the selection criteria^9^ (online supplementary *Table* *S1*) were enrolled in the trial. Study participants were randomized in a 1:1 ratio to double‐blind treatment with either digitoxin (0.05, 0.07, or 0.1 mg) or a placebo, administered orally once daily. For dose titration digitoxin serum concentrations were determined in a blinded fashion for all patients randomized to digitoxin or placebo 6 weeks after randomization in a core laboratory, and dose adjustments were centrally initiated to avoid unblinding. Initiation of digitoxin treatment with the daily maintenance dose and without loading produces steady state levels not until 3–4 weeks.^12^, ^13^ Therefore, to ensure stable digitoxin serum concentrations for standardized dose titration, 6 weeks after start of treatment was chosen as the point of time for determination of digitoxin serum concentrations. In the digitoxin group, dose adjustment employed a pre‐defined algorithm. If digitoxin serum levels were outside the target range of 10.5 to 23.6 nmol/L, doses were reduced or increased to 0.05 or 0.1 mg digitoxin once daily, accordingly. Otherwise, the starting dose of 0.07 mg digitoxin once daily was maintained. In the placebo group, dose adjustment was randomly assigned. Within the standardized dosing protocol, patients did not receive additional loading doses per‐protocol during the trial. More details and rationale for digitoxin dosing are provided in the study protocol and previous publications.^9^, ^10^, ^14^ Randomization was stratified by sex (male or female), NYHA class (II, III, or IV), centre, diagnosed atrial fibrillation (yes or no), and pre‐treatment with cardiac glycosides (yes or no) at randomization (since 6 February 2017, protocol amendment 2.0). Main inclusion criteria for DIGIT‐HF are presented in online supplementary *Table* *S1*. A comprehensive list of the inclusion and exclusion criteria is provided in the design paper.^9^

### Treatment

All patients received SOC in accordance with the 2012 European Society of Cardiology (ESC) HF guidelines at the commencement of the trial.^6^ SOC encompassed the administration of beta‐blockers, ACEi/angiotensin receptor blockers (ARB), MRA, as well as, if indicated, CRT, ICD, and ivabradine upon discretion of the treating physician. SOC was extended by the treatment with ARNI and SGLT2i due to updates of the ESC HF guidelines in 2016 and 2021 during the trial period.^7^, ^8^

### Outcomes and statistical considerations

The primary objective of DIGIT‐HF is to demonstrate the superiority of digitoxin over placebo on top of SOC in HFrEF patients. Superiority is to be concluded if digitoxin improves the composite primary endpoint of time to first hospitalization for worsening HF and all‐cause death (whichever occurs first) to a greater extent than placebo in a time‐to‐event analysis. The key secondary objectives are to test whether digitoxin is (i) non‐inferior regarding the time to all‐cause death and (ii) superior regarding the (rate of) recurrent hospital admissions for worsening HF and all‐cause death. No multiplicity adjustment is needed because the primary and key secondary endpoints are hierarchically tested in the formerly described order. If all confirmatory hypotheses of the main study have been successfully rejected, substudies are also assessed confirmatory in a pre‐defined order. The initial statistical considerations were described in the design paper.^9^ Substantial amendments with regard to the sample size and statistical analyses, and the statistical analysis plan were recently published in a research letter.^10^ Due to the expiration of the augmented and protracted financial support from the Federal Ministry of Education and Research, Germany, patient recruitment was concluded on 30 September 2023. The follow‐up period ended on 29 November 2024, encompassing a total of 1240 randomized patients.

### Comparator trials

We compared baseline characteristics of DIGIT‐HF patients with those in other recent, large, randomized controlled trials, demonstrating improved outcomes in chronic HFrEF, specifically PARADIGM‐HF,^15^ DAPA‐HF,^16^ and EMPEROR‐Reduced.^17^ The respective inclusion and exclusion criteria are summarized in online supplementary *Table* *S1*. Main baseline characteristics are presented as pooled means and standard deviations or absolute and relative frequencies (if provided). Calculations are based on published baseline data of the analysis populations from the aforementioned trials, if not indicated otherwise.

## Results

### Main trial criteria

The main inclusion criteria (online supplementary *Table* *S1*) were very similar in DIGIT‐HF, PARADIGM‐HF, DAPA‐HF, and EMPEROR‐Reduced, as they all included adult patients with symptomatic (NYHA functional class II–IV) chronic HF and a LVEF ≤40%. DIGIT‐HF specified these inclusion criteria to NYHA functional class II and LVEF ≤30%, or NYHA functional class III–IV and LVEF ≤40%. In contrast to PARADIGM‐HF, DAPA‐HF, and EMPEROR‐Reduced, DIGIT‐HF did not require fulfilled B‐type natriuretic peptide (BNP)/N‐terminal proBNP (NT‐proBNP) and renal function (estimated glomerular filtration rate [eGFR]) criteria for patient inclusion. Only PARADIGM‐HF stipulated the necessity of HF hospitalization events within the last 12 month before recruitment, provided natriuretic peptide levels did not exceed pre‐defined values. PARADIGM‐HF requested treatment with beta‐blockers and ACEi/ARB at enrolment only. DAPA‐HF requested medications as recommended by guidelines including beta‐blocker, ACEi/ARB, ARNI, and MRA, the latter if considered by the treating physician. In contrast, EMPEROR‐Reduced requested a medical and device therapy as recommended by guidelines including beta‐blocker, ACEi/ARB/ARNI, MRA, ivabradine and CRT/ICD, which was extended in DIGIT‐HF by ARNI and SGLT2i after approval in 2016 and 2019, respectively.

### Enrolment of study participants

Between 4 May 2015 and 29 September 2023, 1240 patients were randomized in DIGIT‐HF at 65 sites in three countries (Germany, Austria, and Serbia). Here, we report the baseline characteristics of 1212 patients constituting the intention‐to‐treat (ITT) population, i.e. the primary analysis population, which includes all randomized patients who have taken at least one dose of the study drug (25 randomized patients were excluded from the ITT population because they did not take at least one dose of the study drug; one centre with three randomized patients was excluded from the final analysis and thus the ITT population due to non‐compliance with the quality standards for the conduct of clinical trials). Most patients were enrolled in Germany (*n* = 1078, 89% of the ITT population). In addition, patients were recruited in Serbia (*n* = 101, 8%) and Austria (*n* = 33, 3%).

### Demographic and clinical characteristics at baseline

**Figure 1:**
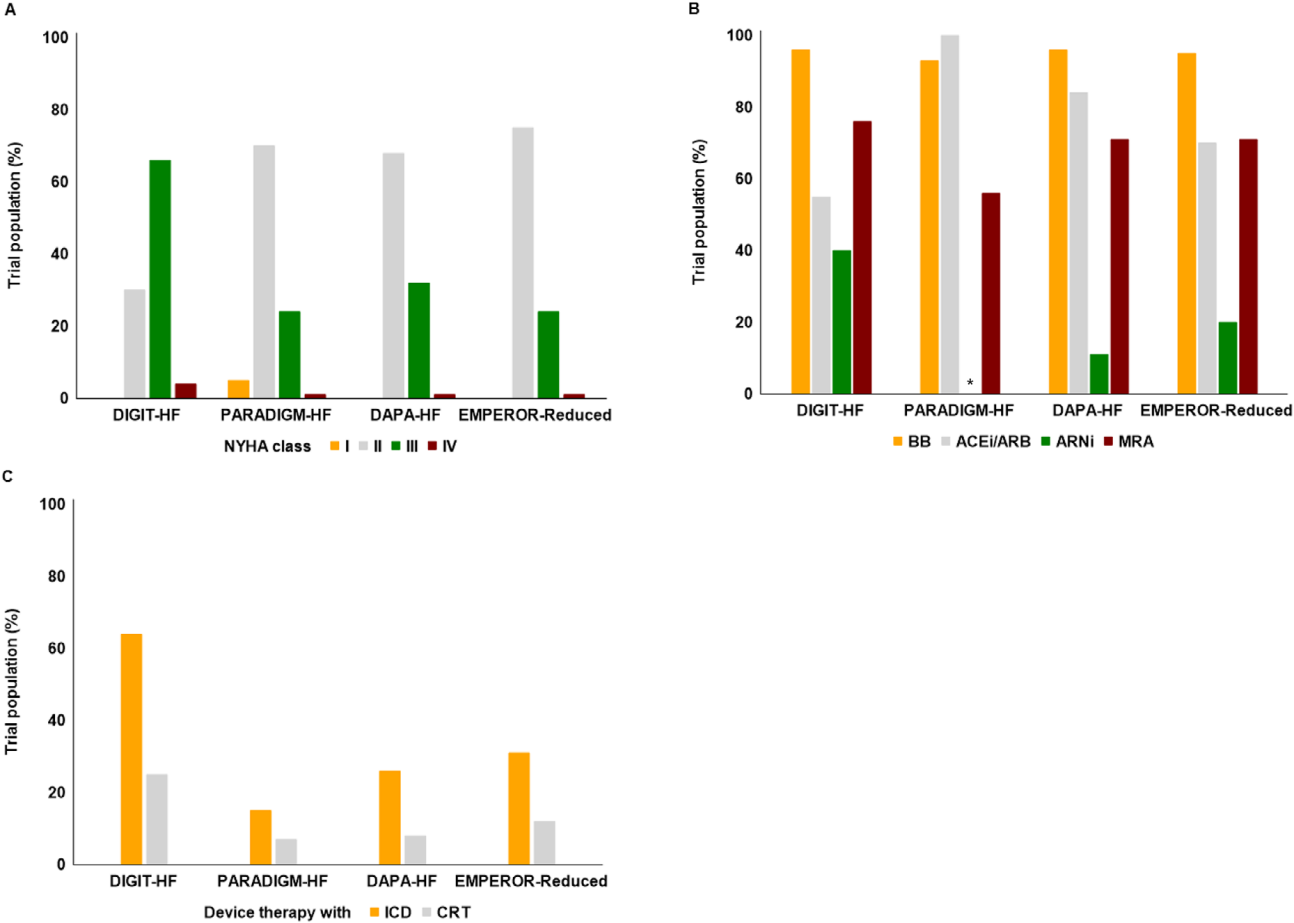
(*A*) New York Heart Association (NYHA) class in % of trial population. (*B*) Concomitant medication in % of trial population. (*C*) Device use in % by trial population. ACEi, angiotensin‐converting enzyme inhibitor; ARB, angiotensin receptor blocker; ARNI, angiotensin receptor–neprilysin inhibitor; BB, beta‐blocker; CRT, cardiac resynchronization therapy; ICD, implantable cardioverter‐defibrillator; MRA, mineralocorticoid receptor antagonist. *In PARADIGM‐HF ~50% of patients were allocated to ARNI per protocol.

*Table* *1* compares the demographics and clinical characteristics of DIGIT‐HF at baseline with PARADIGM‐HF, DAPA‐HF, and EMPEROR‐Reduced.^15^, ^16^, ^17^ The mean age of patients recruited to DIGIT‐HF was 66 ± 11 years, which is comparable to the other trials. The DIGIT‐HF population is predominantly male, with 20% of patients being female, similar to PARADIGM‐HF, DAPA‐HF, and EMPEROR‐Reduced. The majority of patients in DIGIT‐HF were enrolled in Western Europe (Germany/Austria), with a smaller percentage enrolled in Serbia. The mean body mass index of 29 kg/m^2^ was similar in DIGIT‐HF, PARADIGM‐HF, DAPA‐HF, and EMPEROR‐Reduced. In DIGIT‐HF, 30% of patients were classified to NYHA class II and 70% to NYHA classes III–IV, whereas patients in the other HF trials were mainly classified to NYHA class II and less to NYHA classes III–IV (*Figure* *1A*). The median time since diagnosis of HF in DIGIT‐HF was 5 years, confirming the inclusion of patients with chronic HF.

**Table 1:** Baseline characteristics in the DIGIT‐HF, PARADIGM‐HF, DAPA‐HF, and EMPEROR‐Reduced trials

	DIGIT‐HF (*n* = 1212)	PARADIGM‐HF (*n* = 8399)	DAPA‐HF (*n* = 4744)	EMPEROR‐Reduced (*n* = 3730)
**Demographics**				
Age, years	66 ± 11	64 ± 11	66 ± 11	67 ± 11
Female sex, *n* (%)	247 (20)	1832 (22)	1109 (23)	893 (24)
Region, *n* (%)				
Western Europe	1111 (92)	2051 (24)	2154 (45)	1353 (36)
Eastern Europe/Russia/Serbia	101 (8)	2826 (34)		
North America	–	602 (7)	677 (14)	425 (11)
Latin America	–	1433 (17)	817 (17)	1286 (35)
Asia Pacific	–	1487 (18)	1096 (23)	493 (13)
Other	–	–	–	173 (5)
BMI, kg/m^2^	29 ± 6	28 ± 6	28 ± 6	28 ± 5
**HF characteristics**				
NYHA class, *n* (%)				
I	0 (0)	389 (5)	0 (0)	0 (0)
II	359 (30)	5919 (70)	3203 (68)	2800 (75)
III	807 (66)	2018 (24)	1498 (32)	910 (24)
IV	46 (4)	60 (1)	43 (1)	20 (1)
Years since diagnosis of HF, median (IQR)	5 (1–10)	–	–	–
LVEF, %	29 ± 7	29 ± 6	31 ± 7	27 ± 6
LVEF ≤30%, *n* (%)	786 (65)	–	2161 (46)^c^	2729 (73)
NT‐proBNP, pg/ml, median (IQR)	–	1612 (886–3230)	1437 (857–2648)	1907 (1116–3477)
Main cause of HF, *n* (%)				
Ischaemic	633 (52)	5036 (60)	2674 (56)	1929 (52)
Non‐ischaemic/unknown	567 (47)^a^	3363 (40)	2070 (44)	1801 (48)
Blood pressure, mmHg				
Systolic	121 ± 19	122 ± 15	122 ± 16	122 ± 16
Diastolic	73 ± 11	74^b^	74^d^	–
Heart rate, bpm	74 ± 12	73 ± 12	72 ± 12	71 ± 12
Complete LBBB, *n* (%)	244 (20)	– (20)^b^	–	–
QRS duration, ms	127 ± 33	117^b^	–	–

Values are given as mean ± standard deviation, unless otherwise indicated.

BMI, body mass index; IQR, interquartile range; LBBB, left bundle branch block; LVEF, left ventricular ejection fraction; HF, heart failure; IQR, interquartile range; NT‐proBNP, N‐terminal pro‐B‐type natriuretic peptide; NYHA, New York Heart Association.

^a^ Relative frequency does not add to 100% due to missing values (see online supplementary *Table* *S2*).

^b^ Data from publication on specific patients in PARADIGM‐HF, no absolute frequencies or standard deviation can be presented because respective data were not displayed.^18^

^c^ Data from publication on specific patients in DAPA‐HF.^19^

^d^ Data from baseline publication of DAPA‐HF, no standard deviation can be presented because data were not displayed.^20^

The mean LVEF in DIGIT‐HF of 29% is comparable to the other trials. A LVEF of ≤30% was present in 65% of patients in DIGIT‐HF, which is lower compared to EMPEROR‐Reduced but higher than in DAPA‐HF (data for PARADIGM‐HF not provided). Ischaemic heart disease was the predominant cause of HF in 52% of the DIGIT‐HF population, similarly to the other HFrEF trials. The mean systolic and diastolic blood pressures, as well as heart rate, were within the normal range at baseline and comparable across all four trials. Complete left bundle branch block was present at inclusion in 20% of DIGIT‐HF patients, a figure similar to that seen in PARADIGM‐HF (data not provided for DAPA‐HF and EMPEROR‐Reduced).

### Medical history and comorbidities

*Table* *2* provides a comparative analysis of the medical history, comorbidities, and treatment of patients of DIGIT‐HF at baseline in relation to PARADIGM‐HF, DAPA‐HF, and EMPEROR‐Reduced.^15^, ^17^, ^18^, ^21^, ^22^, ^23^ At baseline, 27% of patients in DIGIT‐HF had a history of atrial fibrillation, which is about 10% lower than the rates observed in PARADIGM‐HF, DAPA‐HF, and EMPEROR‐Reduced. Furthermore, 37% of patients in DIGIT‐HF had a history of diabetes mellitus, which is comparable to PARADIGM‐HF, whilst DAPA‐HF and EMPEROR‐Reduced investigating the effects of SGLT2i in HFrEF reported higher rates of patients with diabetes mellitus. The percentage of patients with a history of myocardial infarction was similar in DIGIT‐HF (40%) compared to PARADIGM‐HF and DAPA‐HF, whereas the proportion of patients after myocardial revascularization was higher in DIGIT‐HF (percutaneous coronary intervention 47%, coronary artery bypass grafting 21%) compared to PARADIGM‐HF and DAPA‐HF.

**Table 2:** Medical history, comorbidities, and treatment of patients in the DIGIT‐HF, PARADIGM‐HF, DAPA‐HF, and EMPEROR‐Reduced trials

	DIGIT‐HF (*n* = 1212)	PARADIGM‐HF (*n* = 8399)	DAPA‐HF (*n* = 4744)	EMPEROR‐Reduced (*n* = 3730)
**Medical history and comorbidities**				
Atrial fibrillation, *n* (%)	330 (27)	3091 (37)	1818 (38)	1369 (37)
Obesity, *n* (%)	467 (39)	– (32)^f^	1672 (35)^h^	1167 (31)^j^
Diabetes mellitus, *n* (%)	453 (37)	2907 (35)	1983 (42)	1856 (50)
Hypertension, *n* (%)	960 (79)	5940 (71)	3511 (74)^e^	2698 (72)
Myocardial infarction, *n* (%)	483 (40)	3634 (43)	2088 (44)^e^	–
PCI^a^, *n* (%)	567 (47)	– (21)^f^	1613 (34)^e^	–
CABG, *n* (%)	255 (21)	– (15)^f^	807 (17)^e^	–
Stroke^b^, *n* (%)	117 (10)	725 (9)	4754 (10)^e^	–
Known COPD, *n* (%)	172 (14)	1080 (13)^g^	585 (12)^i^	–
eGFR <60 ml/min/1.73 m^2^, *n* (%)	520 (43)	– (37)^f^	1926 (41)	1799 (48)
Haemoglobin, g/L	137 ± 18	140^e^	136^h^	‐
Anaemia, *n* (%)				
Men	257 (27)	– (21)^e^	– (28)^e^	‐
Women	49 (20)	– (18)^e^	– (26)^e^	‐
**Treatment, *n* (%)**				
Beta‐blocker	1160 (96)	7811 (93)	4558 (96)	3533 (95)
ACEi	435 (36)*	– (77)^f^	2661 (56)	2600 (70)
ARB^c^	228 (19)*	– (22)^f^	1307 (28)	
ARNI	479 (40)	–	508 (11)	727 (20)
MRA	924 (76)	4671 (56)	3370 (71)	2661 (71)
SGLT2i^d^	234 (19)	–	–	–
Ivabradine	106 (9)	– (2)^e^	238 (5)^e^	–
Diuretic	1053 (87)	6738 (80)	4433 (93)	3174 (85)^k^
Cardiac glycosides	9 (1)	2539 (30)	887 (19)	‐
ICD	486 (40)	1243 (15)	1242 (26)	1171 (31)
CRT‐D	293 (24)	574 (7)^l^	354 (8)^l^	442 (12)^l^
CRT‐P	13 (1)			

ACEi, angiotensin‐converting enzyme inhibitor; ARB, angiotensin receptor blocker; ARNI, angiotensin receptor–neprilysin inhibitor; CABG, coronary artery bypass grafting; COPD, chronic obstructive pulmonary disease; CRT‐D, cardiac resynchronization therapy‐defibrillator; CRT‐P, cardiac resynchronization therapy‐pacemaker; eGFR, estimated glomerular filtration rate; ICD, implantable cardioverter‐defibrillator; MRA, mineralocorticoid receptor antagonist; PCI, percutaneous coronary intervention; SGLT2i, sodium–glucose cotransporter 2 inhibitor.

^a^ DIGIT‐HF: PCI/stent.

^b^ DIGIT‐HF: known cerebrovascular disease (e.g. stroke, transient ischaemic attack).

^c^ DIGIT‐HF: AT_1_‐receptor blocker.

^d^ DIGIT‐HF: information on SGLT2i available in electronic case report form after 1 December 2019.

^e^ Data from baseline publication of DAPA‐HF, therefore absolute frequency or standard deviation is only partly available.^20^

^f^ Data from baseline publication of PARADIGM‐HF (pre‐enrolment), therefore no absolute frequency or standard deviation can be presented because it was not displayed.^18^

^g^ Data from publication on specific patients in PARADIGM‐HF.^21^

^h^ Data from the pre‐specified subgroup analysis of the main publication of DAPA‐HF.^15^

^i^ Data from publication on specific patients in DAPA‐HF.^22^

^j^ Data from pre‐specified subgroup analysis of the main publication of EMPEROR‐Reduced.^17^

^k^ Data from publication on specific patients in EMPEROR‐Reduced.^23^

^l^ Only data for CRT device therapy provided without differentiation between CRT‐D and CRT‐P.

* One patient in DIGIT‐HF was treated with an ACEi and ARB at baseline.

The proportion of patients after stroke and with chronic obstructive pulmonary disease was similar in DIGIT‐HF, PARADIGM‐HF, and DAPA‐HF (~10%). The mean eGFR at baseline was comparable in DIGIT‐HF, PARADIGM‐HF, DAPA‐HF, and EMPEROR‐Reduced (~65 ml/min/1.73 m^2^), with a similar proportion of patients having an eGFR <60 ml/min/1.73 m^2^ (~40%). Haemoglobin levels were similar in DIGIT‐HF, PARADIGM‐HF, and DAPA‐HF as well as the portion of men and women with anaemia (~25%).

### Drug and device therapy at baseline

In DIGIT‐HF, beta‐blocker treatment was administered to nearly all patients at baseline (96%), a figure comparable to the other trials. Furthermore, treatment rates with inhibitors of the renin–angiotensin system (ACEi, ARB, ARNI) were very high (>90%) in all the trials (*Table* *2*, *Figure* *1B*). In this regard, a much higher portion of patients received ARNI (40%) at trial entry in DIGIT‐HF compared to DAPA‐HF and EMPEROR‐Reduced; in PARADIGM‐HF ~50% of the trial population was randomized to treatment with ARNI per protocol. MRA prescription rates at baseline were high in DIGIT‐HF (76%), which is comparable to the SGLT2i trials DAPA‐HF and EMPEROR‐Reduced, but higher than in PARADIGM‐HF. Treatment with SGLT2i was present in 19% of patients enrolled into DIGIT‐HF at baseline, whereas SGLT2i were not approved for medical treatment during the conduct of PARADIGM‐HF, but were allocated per protocol to ~50% of the trial populations in DAPA‐HF and EMPEROR‐Reduced. Use of ivabradine at baseline was low in DIGIT‐HF (9%), yet it was higher in comparison to PARADIGM‐HF and DAPA‐HF (data not provided for EMPEROR‐Reduced). A high percentage of the DIGIT‐HF population received diuretics (87%) comparable to the other trials. Very few patients in DIGIT‐HF were pre‐treated with cardiac glycosides (1%) compared to PARADIGM‐HF and DAPA‐HF (data for EMPEROR‐Reduced not provided). The proportion of patients with ICD (64%) and CRT (25%) device therapies was high in DIGIT‐HF, particularly if compared to PARADIGM‐HF and DAPA‐HF but also if compared to EMPEROR‐Reduced (*Figure* *1C*).

### Further detailed information of baseline data

More detailed information on patient history, clinical characteristics, treatment, and laboratory parameters at baseline as well as the number of randomized patients per study site in DIGIT‐HF are presented in online supplementary *Tables* *S2* and *S3*.

## Discussion

The DIGIT‐HF trial represents a significant development in the field of HF research, as it is the first of two large trials currently evaluating the impact of cardiac glycosides on outcomes in HF patients treated with contemporary guideline‐directed HF therapy. This is of particular importance as the DIG trial – conducted more than 30 years ago – investigated the effects of digoxin in the context of a background HF therapy that primarily consisted of ACEi and diuretics.^1^ DIGIT‐HF is a multinational trial (Germany, Austria, Serbia) evaluating digitoxin in symptomatic HF patients with an LVEF ≤40%,^9^ whereas the DECISION trial is conducted in the Netherlands and investigates digoxin in symptomatic HF patients with a LVEF <50% (HF with reduced or mildly reduced ejection fraction).^24^

### Clinical characteristics in DIGIT‐HF and comparator trials

DIGIT‐HF was designed to recruit patients with chronic HFrEF into a simple trial design, with the objective of facilitating transferability to the ‘real world’. As such, the trial was not subject to selection criteria such as natriuretic peptide concentrations or recent HF hospitalizations. As natriuretic peptide determination was (and often is still) not standard in patients with HF in many countries, our design enables application of the results also in low‐ and medium‐income countries where digitalis is often used in HFrEF in clinical practice. However, to ensure the enrichment of a higher risk population inclusion criteria with combination of NYHA II/LVEF <30% and NYHA III/LVEF <40% were chosen. Digitoxin was selected over digoxin due to its advantageous pharmacokinetic profile, which results in more stable serum concentrations compared with digoxin, even in patients with advanced renal dysfunction.^11^ Thus, there was no need for renal function inclusion criteria and to repeatedly monitor digitoxin serum concentrations, supporting the simplicity of the trial. Importantly, during the entire trial period no safety signals regarding digitoxin dosing have been reported also supporting the defined dosing protocol and trial design. Effective digitoxin dose adjustment with the defined dosing protocol to enable a simple trial performance was supported by blinded analysis of digitoxin dosing in the first 394 patients enrolled in DIGIT‐HF previously published. This analysis demonstrated the necessity for dose adjustment in ~40% of patients, with the majority (92%) of patients having a dose reduction from 0.07 mg to 0.05 mg digitoxin once daily and only 8% having a dose increase from 0.07 to 0.1 mg digitoxin once daily.^14^ Digitoxin dose–concentration relationships in the overall trial population and associations with trial endpoints will be performed within the final trial analysis. The DIG trial indicated the beneficial effects of digoxin on outcomes especially in the subgroup of patients with LVEF <30% or NYHA class ≥III.^2^ Therefore, the inclusion criteria for DIGIT‐HF were matched to those of the DIG trial: NYHA class II with LVEF ≤30%, or NYHA classes III–IV with LVEF ≤40%. Consequently, the DIGIT‐HF patient population exhibited a higher proportion of patients with a pronounced HF burden (70% in NYHA classes III–IV) compared to the recent HFrEF trials PARADIGM‐HF, DAPA‐HF, and EMPEROR‐Reduced (25–32%), respectively. DIGIT‐HF may therefore close an important evidence gap for the effective treatment of patients with chronic HFrEF and a pronounced HF burden (NYHA classes III–IV). Despite the option to enrol patients up to an LVEF of 40%, a significant proportion of DIGIT‐HF patients had LVEF ≤30% (65%), which is comparable to EMPEROR‐Reduced (73%). The mean LVEF of 30% was similar to other recent HFrEF trials, and the median time from HF diagnosis of 5 years confirms the inclusion of patients with chronic HF.

The DIG trial excluded patients with atrial fibrillation from enrolment, whereas the DIGIT‐HF trial deliberately set out to include HFrEF patients who also had atrial fibrillation, given that this subgroup has a particularly poor prognosis.^25^ Furthermore, it is important to demonstrate the safety of digitoxin in patients with atrial fibrillation and HF, because retrospective analyses of several patient populations have raised concerns about the adverse effects of cardiac glycosides on outcomes.^26^, ^27^ Nevertheless, DIGIT‐HF enrolled a 10% lower proportion of patients with atrial fibrillation at baseline (27%) compared to PARADIGM‐HF, DAPA‐HF, and EMPEROR‐Reduced (37–38%). This discrepancy can be attributed to the fact that many patients with atrial fibrillation could not be included in DIGIT‐HF due to their utilization of cardiac glycosides for rate control, which precluded their participation in randomization to placebo.

The higher proportion of patients with a history of myocardial revascularizations (percutaneous coronary intervention/coronary artery bypass grafting) in DIGIT‐HF supports the enrolment of a patient population with more pronounced disease compared to PARADIGM‐HF, DAPA‐HF, and EMPEROR‐Reduced. Comorbidities and cardiovascular risk factors in DIGIT‐HF were comparable to PARADIGM‐HF, DAPA‐HF, and EMPEROR‐Reduced with a high prevalence of hypertension (~75%), obesity (~35%) and diabetes mellitus (~40%). However, the proportion of patients with diabetes was highest in the trials investigating the effects of SGLT2i in HFrEF (DAPA‐HF: 42%, EMPEROR‐Reduced: 50%). This is most likely a result of the trial protocols ensuring the enrolment of a certain percentage of patients with diabetes.^16^, ^17^ Due to the pharmacokinetic profile of digitoxin,^11^ DIGIT‐HF omitted implementing stringent selection criteria for renal function parameters; only patients with concomitant severe kidney and liver disease were excluded. Therefore, patients with more impaired renal function were expected to be recruited in DIGIT‐HF compared to other HFrEF trials. However, renal function was similar in DIGIT‐HF compared to PARADIGM‐HF, DAPA‐HF, and EMPEROR‐Reduced with an average eGFR of ~65 mL/min/1.73 m^2^ and ~40% of patients showing an eGFR of <60 ml/min/1.73 m^2^. Depending on the final trial results of DIGIT‐HF and DECISION, digitoxin could be an important treatment option, especially in patients with progressive renal dysfunction or kidney failure, for whom therapy and/or dose escalation with renin–angiotensin–aldosterone system inhibitors, ARNI, SGLT2i is problematic or no option. In these patient group, digitoxin appears to be preferable over digoxin because of its compensatory entero‐hepatic elimination, whereas digoxin may hit toxic concentrations due to its mainly renal excretion.

### Background heart failure therapy in DIGIT‐HF and comparator trials

A very high percentage (~95%) of the populations of DIGIT‐HF, PARADIGM‐HF, DAPA‐HF, and EMPEROR‐Reduced were pretreated with beta‐blockers and inhibitors of the renin–angiotensin system. Notably, at study start, the DIGIT‐HF population showed the highest treatment rate with MRAs (76%) compared to the other trials. During the trial period, treatment with ARNI (2016) and SGLT2i (2019) was approved based on evidence from PARADIGM‐HF and DAPA‐HF/EMPEROR‐Reduced, respectively.^15^, ^16^, ^17^ In DIGIT‐HF, treatment with ARNI (40%) was more common compared to the SGLT2i trials (in PARADIGM‐HF ~50% of patients were allocated to ARNI per protocol) as well as treatment with SGLT2i (19%), which was not present in PARADIGM‐HF and was 50% according to protocol in DAPA‐HF and EMPEROR‐Reduced. A much higher portion of patients in DIGIT‐HF were pretreated with ivabradine (~10%) in comparison to the other trials (max 5%). Interestingly, the higher treatment rate of ivabradine in DIGIT‐HF was not reflected by a reduced heart rate at baseline compared to the other trials despite similar rates of beta‐blocker treatment. The higher treatment rates of ivabradine might be indicative of a HF population with more pronounced disease enrolled in DIGIT‐HF, with limitations in up‐titrating beta‐blockers to HF target doses due to low blood pressures or severe haemodynamic impairment (e.g. 4% of patients in NYHA class IV). This could account for the absence of heart rate reduction with more frequent ivabradine treatment despite similar proportions in beta‐blocker treatment. However, pre‐specified analyses of changes in the prescription rates and dosages of HF medications itself within the trial period will elucidate potential effects on primary and secondary endpoints. Whereas prescription rates of cardiac glycosides were still substantial in the comparator trials (20–30%), these were unexpectedly low in patients included in DIGIT‐HF (1%). This probably is explained by the fact that overall prescription rates of cardiac glycosides for the treatment of HF have steadily decreased, currently cardiac glycosides are primarily prescribed for rate control of atrial fibrillation, and the latter patient population is precluded from DIGIT‐HF. Despite this, the portion of patients with atrial fibrillation recruited to DIGIT‐HF is still substantial and it is important to demonstrate potential effects of digitoxin within the pre‐specified subgroup analysis comparing patients with and without atrial fibrillation.

Two‐thirds of the DIGIT‐HF population is treated with an ICD and 25% with a CRT device therapy. This represents the highest rate of ICD/CRT device therapies reported to date in any HFrEF trial investigating pharmaceutical therapies, including PARADIGM‐HF, DAPA‐HF, and EMPEROR‐Reduced, although the proportion of patients with ICD/CRT devices in these trials has been steadily increasing (PARADIGM‐HF < DAPA‐HF < EMPEROR‐Reduced).

Although prescription of background HF pharmacotherapies in PARADIGM‐HF, DAPA‐HF, EMPEROR‐Reduced, and DIGIT‐HF was high and largely similar, DIGIT‐HF overall represents the most adherent implementation of a guideline‐directed HF therapy at trial entry including ARNI, SGLT2i, ivabradine, and in particular ICD/CRT device therapies. It will be important to observe if this will be reflected by better outcomes compared to other HFrEF trials, or if this might be counterbalanced by the recruitment of a patient population with a more pronounced HF symptom burden.

Some limitations should be addressed while interpreting the baseline data of DIGIT‐HF. A main challenge was the lower than expected recruitment, which was probably due to the initial overestimation of the number of patients to be recruited per trial site. Therefore, the number of trial sites was increased from initially 40 to finally 65. This included extension of the trial to motivated investigators in Austria and Serbia.^10^, ^28^ In addition, competing clinical trials recruiting HFrEF populations funded by pharmaceutical companies (e.g. DAPA‐HF, EMPEROR‐Reduced)^16^, ^17^ started during the DIGIT‐HF‐trial period, which probably impaired recruitment into DIGIT‐HF as well.

The COVID‐19 pandemic also affected recruitment because screening/baseline visits intended to be performed in presence could not be scheduled. Furthermore, trial extension to Serbia was strongly delayed by the COVID‐19 pandemic. However, to explore potential impact of COVID‐19 on the study results, COVID‐related data (e.g. respiratory infections, vaccinations) have been collected and will be analysed.

Omitting criteria for enrichment of a high‐risk HF population such as BNP/NT‐proBNP levels or events due to worsening of HF within the last 12 month before recruitment may reduce the number of relevant endpoint events and therefore the power of the trial. However, factors favouring the simplicity and recruitment of the trial as well as transferability to a real‐world population were weighed to be of more importance when designing the trial. Alternatively, other criteria ensuring inclusion of a high‐risk population were defined such as LVEF ≤30% in NYHA class II and LVEF ≤40% in NYHA class III.

The assumed digitoxin target serum levels and a simplified dosing protocol without frequent controls of digitoxin serum levels may affect a potential treatment effect. However, analysis of blinded digitoxin dose–concentration relationships^14^ as well as the lack of any safety signal during the trial supported a simplified dosing protocol. Also, the assumed digitoxin target serum concentration was chosen on best evidence from available pharmacokinetic and ‐dynamic data^29^ as well as data from the DIG trial.^1^

In summary, DIGIT‐HF exhibits a patient population with a more pronounced HF symptom burden and superior implementation of contemporary HF treatment, encompassing ARNI, SGLT2i as well as ICD/CRT devices, in comparison to recent HF trials. DIGIT‐HF addresses the efficacy and safety of digitoxin, when administered concomitantly with a well‐implemented HF therapy as recommended by recent guidelines, in a patient population with more pronounced HF (Graphical Abstract).

## Supporting information


**Appendix S1.** Supporting Information.
