# Supplementary material for: DIGitoxin to Improve ouTcomes in patients with advanced chronic Heart Failure (DIGIT‐HF): Baseline characteristics compared to recent randomized controlled heart failure trials
Source: Eur J Heart Fail. 2025 May 19;27(7):1224–33. doi: 10.1002/ejhf.3679 (PMC12370576; doi:10.1002/ejhf.3679)
Supplement: Supplementary file 1 — Appendix S1. Supporting Information. [file EJHF-27-1224-s001.docx]

# **Supplementary Material**

**Supplementary Table 1:** **Main trial criteria of DIGIT-HF compared to PARADIGM-HF, DAPA-HF, and EMPEROR-Reduced**

|  | **DIGIT-HF**  **N = 1212** | **PARADIGM-HF**  **N = 8399** | **DAPA-HF**  **N = 4744** | **EMPEROR-Reduced**  **N = 3730** |
| --- | --- | --- | --- | --- |
| **Main inclusion criteria** |  |  |  |  |
| **Age (years)** | ≥18 | ≥18 | ≥18 | ≥18  ≥20 (for Japan only) |
| **NYHA class** | II and LVEF ≤30%  or  III-IV and LVEF ≤ 40%^r^ | II-IV | II-IV | II-IV |
| **LVEF (%)** | ≤30 and NYHA class II  or  ≤40 and NYHA class III-IV | ≤40^a^ | ≤40^d^ | ≤40 |
| **HF hospitalization** | No | Yes^b^ | No | No |
| **Other** |  |  |  |  |
| **eGFR, ml/min/1.73 m²** | ­ - | ≥30 | ≥30 | ≥20 |
| **Systolic blood pressure, mmHg** | ­ - | ≥95 | ≥95 | <100 |
| **BNP/NT-proBNP level, pg/ml** | ­ - | BNP 150 pg/ml (NT-proBNP ≥ 600 pg/ml) | NT-proBNP ≥ 600pg/ml^e^ ,  if concomitant AF/Afl NT-proBNP ≥ 900 pg/ml | EF ≥36% and ≤40%: NT-proBNP ≥2500 pg/ml for patients without AF or ≥5000 pg/ml for patients with AF  EF ≥31% and ≤35%: NT-proBNP ≥1000 pg/ml for patients without AF or ≥2000 pg/ml for patients with AF  EF ≥31% and ≤35%: NT-proBNP ≥1000 pg/ml for patients without AF or ≥2000 pg/ml for patients with AF  EF ≤30%: NT-proBNP ≥600 pg/ml for patients without AF or ≥1200 pg/ml for patients with AF |
| **Run-in** |  |  |  |  |
| **Placebo/control** | No | Yes | No | No |
| **Active** | No | Yes | No | No |
| **Baseline treatment/therapy** | Medical and device therapy for HF (such as ACEi, ARB, beta-blocker, MRA, ARNI (from 2016), ivabradine, SGLT2i (from 2019), ICD, CRT) as recommended by guidelines | beta-blocker, ACEi/ARB must be included,  MRA as indicated | Medications as recommended by guidelines, ACEi, or ARB, ARNI  and beta-blockers should be included, MRA if considered by treating physician | Medical and device therapy for HF (such as ACEi, ARB, beta-blocker, oral diuretics, MRA, ARNI, ivabradine, ICD, CRT) as recommended by guidelines |
| **Comparison** | SOC + Digitoxin p.o. (0.05, 0.07, or 0.1mg/die)  SOC + Placebo p.o. (corresponding to 0.05, 0.07, or 0.1mg Digitoxin tablets) | Enalapril 10 mg b.i.d.  LCZ 696 200 mg b.i.d. | Dapagliflozin p.o. (5 or 10 mg)  Placebo p.o. (corresponding to 5 or 10 mg Dapagliflozin tablets) | SOC + Empagliflozin p.o. (10 mg)  SOC + Placebo p.o. (corresponding to 10 mg Empagliflozin tablets) |
| **Recruitment period** | 2015-2023 | 2009-2012^c^ | 2017-2018 | 2017-2019 |

NYHA, New York Heart Association; LVEF, left ventricular ejection fraction; HF, heart failure; eGFR, estimated glomerular filtration rate; BNP, brain natriuretic peptide; NT-proBNP, N-terminal proB-type natriuretic peptide; AF, atrial fibrillation; ACEi, angiotensin-converting enzyme inhibitor; ARB, angiotensin receptor blocker, ARNI, angiotensin receptor – neprilysin inhibitor; sodium-glucose co-transporter 2 inhibitor; ICD, implantable cardioverter defibrillator; CRT-D, cardiac resynchronisation therapy - implantable cardioverter defibrillator; SOC, standard of care

^a^ changed to ≤35% December 2010

^b^ and BNP ≥100 pg/mL (or NT-proBNP ≥400 pg/mL) or BNP ≥150 pg/mL (NT proBNP ≥600 pg/mL) if no heart failure hospitalization within 12 months

^c^ the last patient entered the run-in in 2012 but was randomized in 2013.

^d^ determined by echocardiogram, radionuclide ventriculogram, contrast angiography, or cardiac MRI) within the last 12 months before enrolment

^e^ if hospitalized for heart failure within the previous 12 months, NT-proBNP ≥400pg/ml) at enrolment or if concomitant atrial fibrillation or atrial flutter at enrolment, NT-proBNP ≥900 pg/ml (irrespective of history of heart failure hospitalization)

^f^ dose consistent with prevailing local and international CV guidelines, stable for at least one week before visit 1 and during the screening period until visit 2 (randomization), except for diuretics stable for only one week before visit 2 to control symptoms

^g^ in combination with evidence-based heart failure therapy for at least six months at the discretion of the treating physician

^h^ dose adjustment at week 6, and, if indicated at week 12 after the start of treatment (target serum concentration of Digitoxin 10.5-23.6nmol/l). Random dose adjustment to remain blinding in the placebo arm.

**Supplementary Table 2: Baseline characteristics of DIGIT-HF patients**

| **DIGIT-HF**  **N = 1212** | | **Mean ± SD**  **or**  **n (%)** | **Missing values**  **n (%)** |
| --- | --- | --- | --- |
| **Randomization strata and demographics** | Age [years] | 66 ± 11 | 0 |
|  | Sex [female] | 247 (20) | 0 |
|  | Atrial fibrillation [yes] | 330 (27) | 0 |
|  | NYHA functional class |  | 0 |
|  | II | 359 (30) |  |
|  | III | 807 (66) |  |
|  | IV | 46 (4) |  |
|  | Pre-treatment with cardiac glycosides [yes] | 9 (1) | 0 |
|  | Region |  | 0 |
|  | EU | 1111 (92) |  |
|  | Serbia | 101 (8) |  |
| **Cardiac diagnosis** | Cardiomyopathy [yes] | 604 (50) | 13 (1) |
|  | Years since diagnosis of heart failure | 6.8 ± 6.8 | 10 (1) |
|  | Main cause of heart failure |  | 12 (1) |
|  | Hypertension | 58 (5) |  |
|  | Primary valvular heart disease | 2 (27) |  |
|  | Congenital heart disease | 3 (0.2) |  |
|  | Cardiomyopathy | 351 (29) |  |
|  | Not clearly determinable | 64 (5) |  |
|  | Other reason | 64 (5) |  |
|  | Coronary artery disease (CAD) | 633 (52) |  |
|  | Previous myocardial infarction [yes] | 483 (40) | 0 |
|  | Left ventricular ejection fraction ≤ 30% [yes] | 786 (65) | 0 |
| **Cardiac risk factors** | Treated diabetes mellitus [yes] | 453 (37) | 2 (0.2) |
|  | Treated arterial hypertension [yes] | 960 (79) | 2 (0.2) |
|  | Treated hyperlipidemia [yes] | 720 (59) | 6 (0.5) |
|  | Smoker |  | 79 (7) |
|  | No (Never smoked) | 439 (36) |  |
|  | Yes | 228 (19) |  |
|  | Ex-smoker (> 6 months clean) | 466 (38) |  |
|  | Chronic alcohol consumption [yes] | 58 (5) | 45 (4) |
|  | Known peripheral artery disease [yes] | 133 (11) | 5 (0.4) |
|  | Known depression [yes] | 82 (7) | 5 (0.4) |
|  | Known cerebrovascular disease [yes] | 117 (10) | 5 (0.4) |
|  | Known COPD [yes] | 172 (14) | 3 (0.2) |
|  | Known liver cirrhosis [yes] | 6 (0.5) | 3 (0.2) |
|  | Known malignancy [yes] | 89 (7) | 6 (0.5) |
|  | Known hypothyreosis [yes] | 121 (10) | 7 (0.6) |
|  | Known hyperthyreosis [yes] | 41 (3) | 5 (0.4) |
|  | Diagnosed periodontal disease/periodontitis with loss of bone | 0 | |
|  | No | 830 (69) |  |
|  | Yes | 28 (2) |  |
|  | Unknown | 354 (29) |  |
| **Previous cardiovascular intervention** | PCI/Stent [yes] | 567 (47) | 4 (0.3) |
|  | Coronary bypass surgery [yes] | 255 (21) | 4 (0.3) |
|  | Valvular surgery [yes] | 134 (11) | 2 (0.2) |
|  | RV pacemaker (VVI, DDD) [yes] | 62 (5) | 1 (0.1) |
|  | ICD [yes] | 486 (40) | 1 (0.1) |
|  | CRT-D [yes] | 293 (24) | 1 (0.1) |
|  | CRT-P [yes] | 13 (1) | 3 (0.2) |
|  | Cardiac contractility modulation [yes] | 18 (2) | 8 (0.7) |
|  | Vagus stimulation [yes] | 6 (0.5) | 7 (0.6) |
|  | Assist device [yes] | 4 (0.3) | 4 (0.3) |
|  | Heart transplantation [yes] | 1 (0.1) | 2 (0.2) |
|  | Reanimation/defibrillation [yes] | 87 (7) | 7 (0.6) |
| **Concomitant medication** | Beta-blocker [yes] | 1160 (96) | 0 |
|  | ACE-inhibitor [yes] | 435 (36) | 0 |
|  | AT1-receptor blocker [yes] | 228 (19) | 0 |
|  | ARNI [yes] | 479 (40) | 0 |
|  | MRA [yes] | 924 (76) | 0 |
|  | SGLT-2 inhibitor [yes]^a^ | 234 (19) | 0 |
|  | Ivabradine [yes] | 106 (9) | 0 |
|  | Diuretic [yes] | 1053 (87) | 0 |
|  | Loop diuretic [yes] | 992 (82) | 0 |
|  | Thiazide diuretic [yes] | 158 (13) | 0 |
|  | Other diuretic [yes] | 99 (8) | 0 |
| **Laboratory parameters** | Hemoglobin [g/dl] | 13.7 ± 1.8 | 3 (0.2) |
|  | Anemia [yes] |  | |
|  | Men | 257 (27) | 3 (0.2) |
|  | Women | 49 (20) | 0 |
|  | Leucocytes [Tsd/µL] | 7.8 ± 2.2 | 3 (0.2) |
|  | Creatinine [µmol/l] | 116.2 ± 52.2 | 1 (0.1) |
|  | eGFR [ml/min/1.73 m^2^] (CKD-EPI) | 65.1 ± 23.3 | 1 (0.1) |
|  | eGFR < 60 ml/min/1.73 m2 | 520 (43) | 1 (0.1) |
|  | Urea [mmol/l] | 8.9 ± 5.1 | 42 (0.4) |
|  | Sodium [mmol/l] | 139.7 ± 3.1 | 4 (0.3) |
|  | Potassium [mmol/l] | 4.5 ± 0.5 | 2 (0.2) |
|  | ASAT [U/l] | 28.9 ± 26.8 | 41 (3) |
|  | ALAT [U/l] | 28.6 ± 35.1 | 28 (2) |
|  | gamma-glutamat-transferaseT [U/l] | 72.4 ± 109.7 | 33 (3) |

SD, standard deviation; NYHA, New York Heart Association; COPD, chronic obstructive lung disease; PCI, percutaneous coronary intervention; RV, reight ventricle, ICD, implantable cardioverter defibrillator; CRT-D, cardiac resynchronisation therapy - implantable cardioverter defibrillator; CRT-P, cardiac resynchronisation therapy -pacemaker; ARNI, angiotensin receptor – neprilysin inhibitor; SGLT2, sodium-glucose co-transporter 2; GFR, glomerular filtration rate; ASAT, aspartate-aminotransferase, ALAT, alanine-aminotransferase

^a^ DIGIT-HF: Information on SGLT-2 inhibitor available in eCRF after 1^st^ Dec. 2019

**Supplementary Table 3: Number of randomised patients by study site**

| **Study Site** | **n (%)** |
| --- | --- |
| Total | 1212 (100%) |
| 1 | 179 (14.8) |
| 2 | 14 (1.2) |
| 4 | 76 (6.3) |
| 8 | 15 (1.2) |
| 10 | 26 (2.1) |
| 11 | 38 (3.1) |
| 13 | 10 (0.8) |
| 15 | 22 (1.8) |
| 16 | 10 (0.8) |
| 19 | 12 (1.0) |
| 24 | 21 (1.7) |
| 25 | 12 (1.0) |
| 26 | 20 (1.7) |
| 28 | 13 (1.1) |
| 33 | 21 (1.7) |
| 36 | 10 (0.8) |
| 38 | 190 (15.7) |
| 41 | 30 (2.5) |
| 45 | 33 (2.7) |
| 46 | 67 (5.5) |
| 48 | 13 (1.1) |
| 52 | 10 (0.8) |
| 59 | 17 (1.4) |
| 65 | 18 (1.5) |
| 68 | 60 (5.0) |
| 69 | 15 (1.2) |
| 71 | 22 (1.8) |
| 74 | 20 (1.7) |
| 75 | 13 (1.1) |
| 77 | 12 (1.0) |
| Pool Serbia* | 101 (8.3) |
| Pool 1* | 21 (1.7) |
| Pool 2* | 22 (1.8) |
| Pool 3* | 24 (2.0) |
| Pool 4* | 25 (2.1) |

* Study sites with a number of randomised patients < 10 were pooled (pooling was performed blinded).

## **List of DIGIT-HF Committees and Investigators**

### **Principal Investigator and Sponsor**

| **Name (role)** | **Department / Institution** |
| --- | --- |
| Prof. Dr. med. Udo Bavendiek (head of study) | Klinik für Kardiologie und Angiologie / Medizinische Hochschule Hannover |
| Prof. Dr. med. Johann Bauersachs (head of study) | Klinik für Kardiologie und Angiologie / Medizinische Hochschule Hannover |
| Prof. Dr. med. Christoph Schindler (sponsors representative) | ZKS, Zentrum für klinische Studien im Namen vom Sponsor Medizinische Hochschule Hannover |
| Prof. Dr. Armin Koch (statistician) | Institut für Biometrie / Medizinische Hochschule Hannover |
| Prof. Dr. med. Dirk O. Stichtenoth (phamracovigilance) | Institut für Klinische Pharmakologie / Medizinische Hochschule Hannover |

### **Trial Steering Committee (TSC)**

| **Name (role)** | **Department / Institution** |
| --- | --- |
| Prof. Dr. med. Udo Bavendiek (head of study) | Klinik für Kardiologie und Angiologie / Medizinische Hochschule Hannover |
| Prof. Dr. med. Johann Bauersachs (head of study) | Klinik für Kardiologie und Angiologie / Medizinische Hochschule Hannover |
| Prof. Dr. Christian Veltmann (medical expert) | Elektrophysiologie Bremen / Klinikum Links der Weser |
| Prof. Dr. Michael Böhm (medical expert) | Klinik für Innere Medizin III – Kardiologie, Angiologie und internistische Intensivmedizin / Universitätsklinikum des Saarlandes |
| Prof. Dr. Armin Koch (statistician of TSC) | Institut für Biometrie / Medizinische Hochschule Hannover |
| Prof. Dr. Heiko von der Leyen (medical) | Außerordentlicher Professor an der Medizinischen Hochschule Hannover |
| Prof. Dr. Stefan Störk (medical expert) | Deutsches Zentrum für Herzinsuffizienz / Universitätsklinikum Würzburg |

### **Data Monitoring Committee (DMC)**

| **Name (role)** | **Department / Institution** |
| --- | --- |
| Prof. Dr. Dr. Stefan D. Anker (head of DMC) | (Gewebe-) Homöostase in Kardiologie und Stoffwechsel – Medizinische Klinik Kardiologie Charité – Universitätsmedizin Berlin |
| Prof. Dr. rer. nat. Hans J. Trampisch (statistician of DMC) | Abteilung für medizinische Informatik, Biometrie und Epidemiologie / Ruhr-Universität Bochum |
| Prof. Dr. Paul Mohacsi (medical expert of DMC) | HerzGefäßZentrum Im Park / HIRSLANDEN Klinik im Park |
| Univ.-Doz. Dr. Gerhard Pölzl (medical expert of DMC) | Universitätsklinik für Innere Medizin III –Kardiologie und Angiologie  Interdisziplinäres Herzinsuffizienzzentrum Tirol – IHZ Zentrum für seltene Herzmuskelerkrankungen / Medizinische Universität Innsbruck |
| Dr. Anika Großhennig (independent statistician) | Institut für Biometrie / Medizinische Hochschule Hannover |
| Maria von Karpowitz (independent statistician) | Institut für Biometrie / Medizinische Hochschule Hannover |

### **Clinical Event Adjudication Committee (CEAC)**

| **Name** | **Department / Institution** |
| --- | --- |
| Prof. Dr. med. Ulrich Tebbe | Institut Klinische Forschung GmbH/ Georg-August-Universität Göttingen |
| Prof. Dr. med. Markus Haass | Abteilung für Kardiologie, Angiologie und internistische Intensivmedizin/ Theresienkrankenhaus Mannheim |
| PD Dr. Dr. med. Stephan von Haehling | Klinik für Kardiologie und Pneumologie/ Universitätsmedizin Göttingen |

### **Principal Investigators at the Study Sites**

| **Name** | **Department / Institution** |
| --- | --- |
| Dr. med. Robert Stöhr (PI), Prof. Dr. med. Nikolaus Marx (Co-PI) | Klinik für Kardiologie, Pneumologie, Angiologie und Internistische Intensivmedizin/ Medizinische Klinik I, Uniklinik RWTH |
| Dr. med. Andreas Rieth (PI), Prof. Dr. med. Veselin Mitrovic (Co-PI) | Abteilung für Kardiologie, Kerckhoff-Klinik Bad Nauheim |
| Dr. med. Andreas Ritzel (PI), Dr. med. Johannes Haas (CO-PI) | Gemeinschaftspraxis für Herz und Lunge  und ambulantes Schlaflabor am Bruderwald, Bamberg |
| Prof. Dr. med. Frank Edelmann (PI), Dr. Tobias Trippel (Co-PI) | Charité Campus Virchow Kinikum Berlin, Medizinische Klinik für Kardiologie |
| Dr. med. Sebastian Winkler (PI), Dr. med. Mirko Seidel (Co-PI) | Unfallkrankenhaus Berlin, Klinik für Innere Medizin |
| Dr. med. Monika Ernst (PI), Prof. Dr. med. Christoph Hanefeld (Co-PI) | St. Elisabeth-Hospital, Medizinische Klinik III, Bochum |
| Prof. Dr. med. Andreas Mügge (PI), Dr. Kristina Becker (Co-PI) | St. Josef-Hospital, Medizinische Klinik II - Klinik für Kardiologie, Bochum |
| Prof. Dr. med. Georg Nickenig (PI), Dr. med. Ulrich M. Becher (Co-PI) | Universitätsklinikum Bonn, Medizinische Klinik und Poliklinik II |
| Prim. Univ. Prof. Dr. Johann Auer (PI) | Krankenhaus St. Josef, Abteilung für Innere Medizin 1 mit Kardiologie und int. Intensivmedizin, Braunau |
| Prof. Dr. med. Rainer Hambrecht (PI), Prof. Dr. med. Harm Wienbergen (Co-PI) | Klinikum Links der Weser, Herzzentrum Bremen, Klinik für Kardiologie & Angiologie |
| Prof. Dr. med. Johannes Brachmann (PI), Dr. med. Steffen Schnupp (Co-PI) | Klinikum Coburg, II. Medizinische Klinik, Kardiologie-Angiologie-Pneumologie |
| Dr. med. Ralph Oeckinghaus (PI), Dr. med. Timo Aschenbrenner (Co-PI) | Klinikum Lippe, Kardiologie, Angiologie und Intensivmedizin, Detmold |
| PD Dr. med. Christopher Piorkowski (PI), Dr. med. Thomas Paul Gaspar (Co-PI) | Herzzentrum Dresden GmbH-Universitätsklilnikum, Abteilung Invasive Elektrophysiologie |
| Prof. Dr. med. Christian Meyer (PI), Dr. med Kristin Riße (Co-PI) | Ev. Krankenhaus, Klinik für Kardiologie, Düsseldorf |
| Dr. med. Ralf Westenfeld (PI), Prof. Dr. med. Malte Kelm (Co-PI) | Universitätsklinikum Düsseldorf, Klinik für Kardiologie, Pneumologie und Angiologie |
| Prof. Dr. med. Constantin von zur Mühlen (PI), PD Dr. med. Sebastian Grundmann (Co-PI) | Universitätsklinikum Freiburg, Innere Medizin III Kardiologie und Angiologie |
| Dr. Kristian Hellenkamp (PI), PD Dr. Tim Seidler (Co-PI) | Universitätsmedizin Göttingen, Klinik für Kardiologie und Pneumologie |
| Univ.-Prof. Dr. Friedrich Fruhwald (PI), Dr. med. Klemens Ablasser (Co-PI) | Medizinische Universität Graz, Klinische Abteilung für Kardiologie |
| Dr. med. Alexander Vogt (PI), Dr. med. Sebastian Nuding (Co-PI) | Universitätsklinikum Halle (Saale) |
| Prof. Dr. med. Herbert Nägele (PI), Dr. Daniel Stierle (Co-PI) | Albertinen-Krankenhaus, Department für Herzinsuffizenz und Devicetherapie, Hamburg |
| PD Dr. med. Dirk Westermann (PI), Dr. med. Mahir Karakas (Co-PI) | UKE, Universitäres Herzzentrum Hamburg,  Klinik und Poliklinik für Allg. und Interventionelle Kardiologie |
| Prof. Dr. med. Udo Bavendiek (PI), Prof. Dr. med. Johann Bauersachs (Co-PI) | Medizinische Hochschule Hannover, Klinik für Kardiologie & Angiologie |
| Prof. Dr. med. Lutz Frankenstein (PI), Dr. med. Tobias Täger (Co-PI) | Universitätsklinikum Heidelberg, Innere Medizin III, Kardiologie, Angiologie, Pneumologie |
| Prof. Dr. med. Michael Böhm (PI), Dr. med. Ingrid Kindermann (Co-PI) | Universitätsklinikum des Saarlandes, Klinik für Innere Medizin III, Kardiologie, Angiologie und Internistische Intensivmedizin, Homburg |
| Prof. Dr. med. Paul Christian Schulze (PI), Julian Georg Westphal (Co-PI) | Universitätsklinikum, Klinik für Innere Medizin I Kardiologie, Jena |
| Prof. Dr. med. Roman Pfister (PI), Prof. Dr. med. Stephan Rosenkranz (Co-PI) | Herzzentrum Uniklinik Köln, Klinik III für Innere Medizin |
| Prof. Dr. med. Rolf Wachter (PI), Dr. med. Michael Metze (Co-PI) | Universität Leipzig, Klinik und Poliklinik für Kardiologie |
| PD Dr. Marcus Sandri (PI), Prof. Dr. med. Holger Thiele (Co-PI) | Leipzig Heart Institute GmbH |
| Dr. med. Tobias Graf (PI), Dr. med. Jan-Christian Reil (Co-PI) | Universitätsklinikum Schleswig-Holstein, Medizinische Klinik II - Kardiologie, Angiologie, Intensivmedizin |
| Prof. Dr. med. Rüdiger Braun-Dullaeus (PI), Prof. Dr. med. Alexander Schmeißer (Co-PI) | Universitätsklinikum Magdeburg A. ö. R.  Klinik für Kardiologie, Angiologie und Pneumologie |
| Prof. Dr. med. Thomas Münzel (PI), Prof. Dr. med. Tommaso Gori (Co-PI) | Universitätsmedizin der Johannes Gutenberg-Universität Mainz, 2. Medizinische Klinik und Poliklinik |
| Prof. Dr. med. Bernhard Schieffer (PI), Prof. Dr. med. Wolfram Grimm (Co-PI) | Universitätsklinikum Gießen und Marburg  Standort Marburg, Klinik für Kardiologie |
| Dr. med. Jens Taggeselle (PI), Dr. med. Antje Stumpp (Co-PI) | Internistische Praxis Dr. med. Taggeselle, Markkleeberg |
| Dr. med. Roland Prondzinsky (PI), Dr. Susanne Rode (Co-PI) | Carl von Basedow Klinikum Saalekreis gGmbH  Kardiologie - Angiologie – Diabetologie, Merseburg |
| Dr. Norbert Schön (PI), Dr. med. Brigitte Schön (Co-PI) | Dr. Schön, Kardiologisch-Angiologische Schwerpunktpraxis, Mühldorf am Inn |
| Prof. Dr. med Stefan Kääb (PI), Dr. Stefan Brunner (Co-PI) | Universitätsklinikum München, Campus Innenstadt, Medizinische Klinik und Poliklinik I |
| Dr. med. Johannes Schwab (PI), Prof. Dr. med. Matthias Pauschinger (Co-PI) | Klinikum Nürnberg Süd, Medizinische Klinik 8, Schwerpunkt Kardiologie |
| Prof. Dr. Andreas Götte (PI), Dr. Volker Rickert (Co-PI) | St. Vincenzkrankenhaus GmbH, Medizinische Klinik II – Kardiologie, Paderborn |
| Dr. med. Uwe Gremmler (PI), Michael Krumm (Co-PI) | Kardiologisches Zentrum Peine, MVZ |
| Prof. Dr. med. Lars Maier (PI), Prof. Dr. med. Bernhard Unsöld (Co-PI) | Universitätsklinikum Regensburg, Klinik für Poliklinik für Innere Medizin II |
| Dr. med. Markus Schwefer (PI), Dr. med. Stefan Hettwer (Co-PI) | Elblandklinikum Riesa, Klinik für Innere Medizin I |
| Dr. med. Stefan Rausch (PI), PD Dr. med. Kyrill Rogacev (Co-PI) | MVZ Schwerin West GmbH, Kardiologie |
| PD Dr. med. Sebastian Philipp (PI), Torsten Lauf (Co-PI) | Elbe Kliniken Stade-Buxtehude, Innere Medizin - Kardiologie und Intensivmedizin |
| Univ.-Doz. Dr. Martin Hülsmann (PI) | Medizinische Universität Wien, Univ. Klinik für Innere Medizin II, Abteilung für Kardiologie |
| Prof. Dr. med. Stefan Störk (PI), Dr. med. Bettina Kraus (Co-PI) | Universitätsklinikum Würzburg, Deutsches Zentrum für Herzinsuffizienz |
